# Requirements for a minimum standard of care for phenylketonuria: the patients’ perspective

**DOI:** 10.1186/1750-1172-8-191

**Published:** 2013-12-17

**Authors:** Tobias S Hagedorn, Paul van Berkel, Gregor Hammerschmidt, Markéta Lhotáková, Rosalia Pasqual Saludes

**Affiliations:** 1 European Society for Phenylketonuria and Allied Disorders (E.S.PKU), Melsele, Belgium; 2 Nederlandse Phenylketonurie Vereniging, Bunnik, The Netherlands; 3 Deutsche Interessengemeinschaft Phenylketonurie, Hiddenhausen, Germany; 4 Österreichische Gesellschaft für angeborene Stoffwechselstörungen, Vienna, Austria; 5 National Association for PKU and other inherited metabolic disorders, Prague, Czech Republic; 6 Federación Española de Fenilcetornuria y Otros Trastornos del Metabolismo(FAE PKU Y OTM), Seville, Spain

**Keywords:** Phenylketonuria, Standards of care, Screening, Guidelines, Europe, Centres of Expertise, Healthcare agenda, Patient advocacy, Patient group, Patient voice

## Abstract

Phenylketonuria (PKU, ORPHA716) is an inherited disorder that affects about one in every 10,000 children born in Europe. Early and continuous application of a modified diet is largely successful in preventing the devastating brain damage associated with untreated PKU. The management of PKU is inconsistent: there are few national guidelines, and these tend to be incomplete and implemented sporadically. In this article, the first-ever pan- European patient/carer perspective on optimal PKU care, the European Society for Phenylketonuria and Allied Disorders (E.S.PKU) proposes recommendations for a minimum standard of care for PKU, to underpin the development of new pan-European guideline for the management of PKU. New standards of best practice should guarantee equal access to screening, treatment and monitoring throughout Europe. Screening protocols and interpretation of screening results should be standardised. Experienced Centres of Expertise are required, in line with current European Union policy, to guarantee a defined standard of multidisciplinary treatment and care for all medical and social aspects of PKU. Women of childbearing age require especially intensive management, due to the risk of severe risks to the foetus conferred by uncontrolled PKU. All aspects of treatment should be reimbursed to ensure uniform access across Europe to guideline-driven, evidence-based care. The E.S.PKU urges PKU healthcare professionals caring for people with PKU to take the lead in developing evidence based guidelines on PKU, while continuing to play an active role in serving as the voice of patients and their families, whose lives are affected by the condition.

## Introduction

Phenylketonuria (PKU, ORPHA716) is a rare inherited disorder that affects around one in every 10,000 children born in Europe [1]. The metabolic defect underlying PKU is a mutation in the gene coding for the enzyme, phenylalanine hydroxylase (PAH), which is responsible for the transformation of phenylalanine into tyrosine [2-4]. Impairment of PAH activity in PKU causes increased levels of phenylalanine that if untreated cause devastating damage to the brain, with severe mental disability, reduced IQ, seizures and tremors, impaired executive function, psychological and behavioural issues and social difficulties [4-6].

Most patients with PKU are identified during neonatal screening [6] and all patients then require lifelong treatment [7]. The mainstay of the therapeutic management of PKU is a modified diet that includes specially manufactured foods low in protein, and phenylalanine-free amino acid supplements [3,4]. Maintaining adequate adherence to this diet is challenging, but effective in preventing the severe brain damage associated with uncontrolled blood phenylalanine, and allowing individuals with PKU to lead full and successful lives [5,7-9]. A pharmacologic treatment option, sapropterin, is available for prescription in a growing number of counties [10,11]. A number of other potential treatments that may contribute increasingly to the management of PKU in the future include better and more palatable phenylalanine-free foods, glycomacropeptide (a natural protein free of phenylalanine), large, neutral amino acids, phenylalanine-ammonia lyase (an injectable enzyme that metabolises phenylalanine) and – for the longer term – gene therapy approaches; these have been reviewed elsewhere [12].

Preventing the severely adverse outcomes previously observed in people with PKU is one of the great success stories of modern medicine and PKU must not be given low priority in European healthcare agendas due to its relatively low prevalence: All patients deserve a level of treatment, which allows them to achieve optimal long-term outcomes. Nevertheless, there is considerable variability in clinical practice in PKU centres across Europe [13-15]. In this review, we set out to provide the first pan-European patient perspective on optimal PKU care and provide proposals for a minimum standard of care in the absence of universal European clinical guidelines. In this way, we provide a basis for healthcare professionals to develop evidence-based guidelines in the future.

## Methods

A delegate workshop of the European Society for Phenylketonuria and Allied Disorders (E.S.PKU), involving 21 members from 15 countries, elected a working group (the co-authors) to prepare a consensus paper on best practice for the care of people with PKU, considering the current state and future direction of the management of PKU in Europe. The review is also based on data from the literature, findings from a benchmark report previously developed by the E.S.PKU and presented at the European Parliament in February 2012 (see Table 1) [15], and experiences from patients and carers. The key findings from this report are summarised in Table 1, and these implications of these issues for the future management of PKU are discussed below. For clarity, we include Turkey when considering the management of PKU in Europe throughout this article.

**Table 1:** **Summary of issues common to European countries from a benchmark report, “PKU: Closing the Gaps in Care”, produced by the European Society of PKU and Allied Disorders**[18]

**Issue**	**Key findings**
Neonatal screening	Most countries include blood phenylalanine in neonatal screening
Availability of management guidelines for PKU	Only available for France (2005), Germany (1999), the UK (1993), and Poland (2001)
Target levels for blood phenylalanine	Inconsistent between countries for different age ranges
Composition of healthcare teams	Variable roles for dietician/nutritionist impact on the quality of care
Access to care	Variable access to care, with not all patients are being offered all treatment options that could improve their condition and quality of life
Routine clinical practice	Variable practices for diagnosis and guidance of treatment, Lack of specialist centres
Reimbursement	Variable reimbursement policies for drug and dietary treatment, including amino acid supplements and low protein foods within and across countries
Transition to adult care	Young patients need more support in becoming self-reliant in PKU management
Special low-protein foods	Lack of palatability may hinder adherence to dietary management
Families	The demands of PKU place a strain on family relationships and adolescents may find difficulties associated with PKU in social interaction

### Inconsistent management of PKU in Europe

The management of PKU varies widely across Europe, including different approaches to defining PKU phenotypes, target levels for phenylalanine, and practices for following up patients [13-16]. The E.S.PKU benchmark report (Table 1) found that published national guidelines for the management of PKU are available for only four countries, and none of these has been updated recently [15]. In addition, different countries employ different acceptable ranges for blood phenylalanine levels across age ranges. Phenylalanine thresholds for initiation of dietary management at screening vary from 300–600 μmol/L in different European countries. These differences persist throughout the patient’s life: for example, the upper bound of the target range for 10–12 year olds varied from 240 μmol/L in Turkey to 900 μmol/L in Austria and Germany, with marked differences between countries for all other age ranges [3]. This inconsistency of approach has been confirmed by surveys conducted by expert groups working in this area [13,17].

Limitations in the number of centres and healthcare professionals expert in the management of PKU and differences in the training and responsibilities of healthcare teams possibly contribute to this variability in treatment responses [15,17]. Moreover, the status and roles of dieticians/nutritionists differs between countries (there is no standardised qualification or international association for those managing diet in patients with PKU), as do practices for allocating a daily allowance of phenylalanine (some countries do not), systems for phenylalanine exchanges, and the types of food permitted [17]. Unsurprisingly, the proportion of patients with chronically elevated blood phenylalanine varies markedly across Europe with potentially adverse consequences for long-term neurocognitive outcomes [14].

### Improving the management of PKU in Europe

#### Screening and diagnosis

Neonatal screening for PKU is effective and cost-effective [6,18]. All countries except Finland and Malta (where the prevalence of PKU is low) include phenylalanine in national neonatal screening programmes. Neonatal screening for PKU should be mandatory and available at no cost to every family, to ensure universal coverage of the population. In the UK, for example, screening for PKU is offered free of charge for all newborns at 5 days of age [19], although it is unclear what proportion of parents/carers refuse screening. An audit of the timing and true population coverage of newborn screening across Europe is warranted. Treatment (along with education of carers) should start immediately after confirmation of the diagnosis of PKU, as any delay in treatment exposes infants unnecessarilyto hyperphenylalaninaemia.

The diagnosis of a lifelong genetic condition places a major burden on families [20], and trained personnel should be available to guide families through the nature of PKU, the consequences of the diagnosis for each stage of life and available treatment options [21]. Older individuals may have undiagnosed and consequently untreated PKU, particularly where neonatal screening has been introduced relatively recently [22]. Accordingly, measurement of phenylalanine should be performed in older individuals where signs of mental retardation are consistent with untreated PKU. Genotyping can contribute to diagnosis of the precise PKU phenotype in positive screenees, and should also be made universally available. Each patient should be tested for responsiveness to sapropterin, and each patient shown to be responsive to sapropterin should have access to this treatment. Finally, international registries are important for defining prevalence, assessing the uniformity of practice across regions, surveying standards of care, and as a starting point for future research programmes.

#### Acceptable ranges for blood phenylalanine and other amino acids

An inconsistent approach to the management of blood phenylalanine in PKU is an important barrier to the adoption of uniform European guidelines for this condition, as described above. Although there are some differences on common phenotypes of PKU in Europe (milder forms of HPA/PKU are especially common in Spain, for example [23]), there is no reason to believe that the pathological effects of phenylalanine differ between the new born populations of different European countries. Evidence-based, age-dependent ranges for blood phenylalanine and other amino acids from birth onwards should be applied uniformly across Europe. Specific ranges will be needed for some specific patient groups, however, such as women who are pregnant or considering pregnancy, or late-treated patients who already have neurocognitive sequelae of untreated PKU. It is important to remember in this regard that a “target” or “goal” for phenylalanine, as commonly expressed, really sets an upper limit of the acceptable range for this amino acid. In reality, the patient’s target level should be set individually as the lowest possible safe level, as close as possible to non-PKU levels, but taking into account adherence and other practical issues. Conversely, there is a danger that overzealous management of blood phenylalanine, in the pursuit of blood levels close to those of people without PKU, could lead to levels being too low, and this should be avoided.

Regular testing for blood phenylalanine (at intervals that vary with age) will be necessary to ensure continued optimal control of blood levels of phenylalanine and other amino acids. Members of the healthcare team must be able to provide timely and age-appropriate feedback, and access to a dietician/nutritionist is important to help patients improve their control of phenylalanine levels. Caregivers should also be trained to support the patient through the complex and challenging process of managing their PKU. Self-monitoring of blood phenylalanine has been discussed for some years, based on analogy to diabetes, where immediate information on blood glucoselevels allows patients to adjust their treatment to optimise metabolic control [24]. In principle, a home monitoring kit for blood phenylalanine could be a useful addition to themanagement of a well-motivated, knowledgeable and compliant patient. However, such an approach should never replace regular contact with the treatment centre. Home blood sampling provides a middle ground, where blood phenylalanine results are available more regularly compared with waiting for a clinic visit to have blood phenylalanine measured. A recent randomised trial found that increased access to blood phenylalanine results to facilitate self-management was popular with patients with PKU, but that there was no improvement in blood phenylalanine levels overall, or in the number of out-of-range levels [25]. The true place of home monitoring (or home blood sampling) in the management of PKU has yet to be established. As with other innovations in healthcare delivery, the establishment of a Europe-wide registry will be important to track evolving standards of care.

Although most attention is focussed on the management of blood phenylalanine levels in PKU, as the best validated surrogate marker for risk of damage to the central nervous system, it is also important to ensure that people with PKU maintain adequate intake of amino acids other than phenylalanine [3,4].

#### Importance of multidisciplinary care

The optimal management of PKU is challenging and a variety of healthcare professionals contribute importantly to the care of these patients, including physicians, dieticians/nutritionists and psychologists, with a designated member of the team, who has relevant training and expertise, to act as a central point of contact. In particular, the dietician/nutritionist has a number of key roles within the multidisciplinary team, including gathering information on eating behaviours, to support healthcare decisions. In addition, a paediatrician should initially follow the cognitive development of the child. Care pathways are designed to prevent the development ofcomplications of PKU, particularly neuropsychological deficits. It is important, therefore, that all patients have access to all members of the multidisciplinary team throughout their care, including a psychologist, rather than specialist referral only being arranged when the presence of an adverse outcome is already suspected. Finally, there is a need for adequate resources to prevent excessive case loads diluting access to care.

This multidisciplinary approach is consistent with the requirements of a Centre of Excellence (CE), as defined recently by the European Union Committee of Experts on Rare Diseases [26]. The main requirements for such a Centre of Excellence [27] are summarised below:

•Sufficient professional qualification (certification or accreditation and a number of recognised publications) at both clinical and scientific level, and an agreed commitment to cooperate and share information among professionals;

•An annual Activity Report comparing performance with the preceding year;

•Patient access to a multi-disciplinary team of experts (see above);

•A holistic approach integrating medical and social aspects;

•Combination of research and care with commitment to participate in research activities at European and international level, if appropriate;

•Education, information and communication outreach activities with the public and primary health care professionals;

•Training for health professionals;

•Activities to empower patients and collaboration with patient organisations;

•E-health solutions (e.g. shared case management systems and systems for tele-expertise or online patient communication) shall be considered (with sufficient data protection).

The number of CEs should depend on country-size and population density and offer a good balance between accessibility and experience. European countries outside the European Union (EU) should adopt as many as possible the same criteria for quality of care (although local situations related to, e.g. funding or numbers of patients may render full compliance impractical for some centres, at least in the short term). Transition from the current decentralised care systems to an international, networked system of CEs will take time, and intermediate quality standards may be needed in the interim. The recommendation on CE follows an earlier EU recommendation from 2009, which advises the establishment of an international (PKU) centre for medical education and clinical research within an EU network [27]. Patient advocacy organisations, including the E.S.PKU and its member organisations, have a key role to play in the evolution of a truly international focus on improving standards of care for people with PKU.

#### Education

The challenging nature of following a special diet throughout life (essential for the majority of patients with PKU) places a special emphasis on the need for education. In infancy, the diet of the child is determined entirely by the primary caregiver(s). There is some evidence that the level of blood phenylalanine in a child with PKU varies inversely with the mother’s level of knowledge of dietary management for this condition [28]. The growing child will exert increasing influence on dietary choices, and will experience increasing opportunity to eat prohibited foods while not under the caregiver’s direct control, especially during adolescence (see the section on transition to adult care, below). Accordingly, education of patients, caregivers and other groups involved in the care of the child, such as schools, will be required at appropriate times, using age-appropriate language and materials. Education must be reinforced periodically, as shown by studies where one-off educational interventions initially improved knowledge of dietary management of PKU and/or blood phenylalanine levels, followed by a disappearance of these benefits over a period of months [29,30]. Given the difficulty of maintaining strict dietary control, a relaxation of vigilance over time on the part of patient or caregiver may also be a source of poor control of blood phenylalanine, again requiring reinforcement of the need for and benefit of optimal dietary management. It is important to remember that improving knowledge about the appropriate management of PKU does not always lead to improved compliance, and patients must be motivated to do so [30].

#### Special patient groups

##### Transition from paediatric to adult care

The transition from paediatric to adult care is problematic in PKU, as with other diseases that place a challenging burden of adherence to difficult treatment regimens. Studies have demonstrated poorer control of blood phenylalanine levels in adolescent patients compared with younger patients) [31,32]. Indeed, more complex treatment regimens in general are associated, on average, with poorer outcomes during the transition from paediatric to adult care [33]. Adolescence is a time of emotional turmoil and young people commonly experiment with autonomy and challenge sources of authority at this time. In addition, adolescents with PKU are subject to peer pressure to eat everyday foods with their non-PKU peers in social situations.

It is important to note that, although indices of quality of life and educational or professional achievement are not impaired on average, achieving autonomy and forming mature adult relationships are somewhat delayed in individuals with PKU [34], suggesting that healthcare professionals should address issues additional to control of phenylalanine levels from early on. Careful management of dietary or pharmacologic control of PKU is feasible in adolescents, when these individuals are managed appropriately. Parenting style influences blood phenylalanine levels in young patients with PKU, and parents may need support and counselling to manage the transition of their child with PKU to adult care services [35]. A lack of confidence among generalist physicians in dealing with patients with chronic diseases of childhood origin places emphasis on the need for specialised knowledge of the management of rare inherited diseases, such as PKU, with continuity of care from an expert, multidisciplinary healthcare team [36]. The involvement of a psychologist within the multidisciplinary team is particularly important, especially at times of a change in the patient’s life, to prevent or deal with emotional or psychosocial issues that may impair compliance with therapeutic management of PKU.

The management of adults with PKU is often incompletely understood [36,37]. Only a small number of European centres currently provide healthcare teams with specific expertise in the management of adult patients with PKU. Increasing the ability of centres to manage adult patients would provide an important improvement to the lifelong care of these patients.

##### Women of childbearing age and PKU in pregnancy

Uncontrolled HPA is teratogenic, producing a range of severe cognitive, neurological and physical deficits that resemble those of foetal alcohol syndrome. Damage to the foetus occurs early in PKU, and female patients of childbearing age need to be counselled and educated on the dangers of unplanned pregnancy (coordinated medical and nutritional control and monitoring should be established before *and* during a pregnancy). Available clinical evidence suggests that, for an unplanned pregnancy, establishing control of phenylalanine during the first trimester is essential to protect the developing foetus [38]. Adults with PKU who have been off-diet often find it difficult to resume dietary control [39], and immediate multidisciplinary support is required should a woman with PKU known to be considering a pregnancy. Specific guidelines for more intensive nutritional and metabolic monitoring are required for women considering pregnancy and throughout pregnancy.

##### Patients with PKU not complying with dietary management

Many patients with PKU lose contact with physicians, and resuming dietary adherence is challenging. However, data from adolescents and adults with PKU suggest that resuming adequate control of blood phenylalanine confers a range of benefits related to self-reported general health, psychosocial outcomes (happiness, alertness, impulsivity, calmness, vitality), quality of life and reduced mood swings [39,40]. Studies employing electroencephalography and positron emission tomography have demonstrated improvements in indices of brain function after improved control of blood phenylalanine [41,42]. Patients who discontinue dietary management should be followed up by healthcare professionals [21]. Special foods for PKU have been described by patients as lacking palatability [15], although these foods have improved in recent years [8] and further improvements in these products may help to support better compliance.

Some patients who never received dietary management, with consequent severe cognitive deficit, can be found in care homes in most European countries. These patients should be actively sought out, as dietary management can improve their quality of life [43,44]. Care home staff should be encouraged to work with healthcare professionals in managing these patients.

#### Cost and reimbursement issues

**Figure 1:**
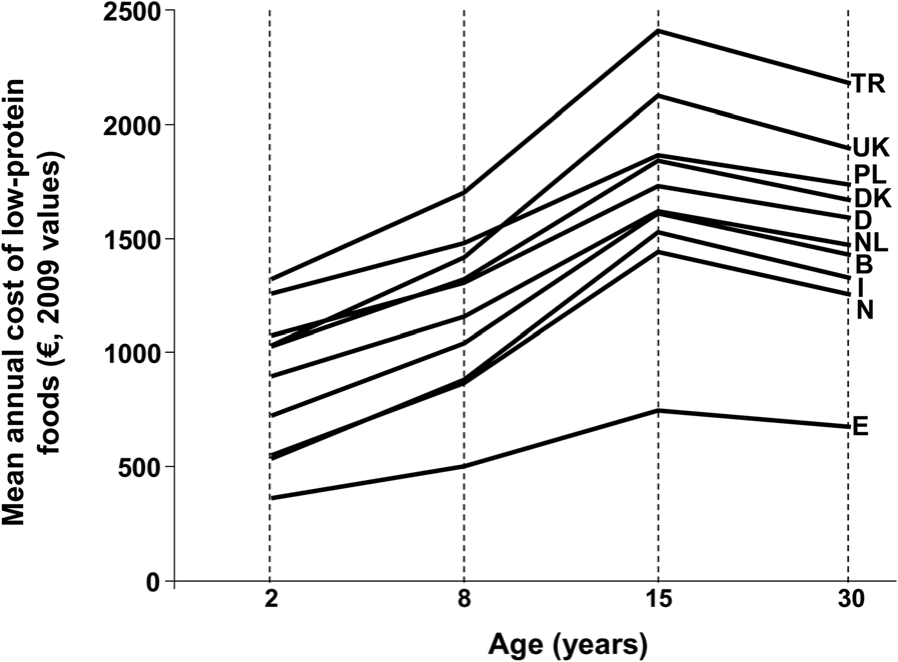
**Chart showing average annual cost of low-protein foods at different ages in 10 European countries.** Drawn from data presented in reference [45]. Assumes 40% of energy intake is from low-protein foods.

Any guideline for the management of PKU will stress the importance of adherence to the special diet, but the requirements for managing the special diet of a patient with PKU places a significant cost burden on families (Figure 1) which will represent a significant barrier to the delivery of evidence-based care [45]. Phenylalanine-free food is an essential therapeutic intervention for people with PKU: this should be accessible to all patients without any obstacles, ideally distributed through the same distribution channels as amino acid supplements (pharmacies) and subsidised fully. Nutritional deficiency can complicate the dietary management of PKU [46], so every patient should be supplied with sufficient supplies of other supplements (micronutrients, fatty acids), as determined by the healthcare team. Indeed, a government subsidy program to finance all treatment options should be considered to increase accessibility to optimal care for patients, and this should be uniform across Europe to support pan-European guidelines. Patients/caregivers should receive appropriate support for adequate amino acid supplements and low protein foods. Providing special foods and supplements themselves, rather than funds to obtain them, may be one means of ensuring that patients receive the correct dietary intervention. Other areas important for reimbursement include outpatient multidisciplinary consultations and treatment (diet and/or drugs), neurocognitive and psychosocial assessment and management, additional diagnostic tests if required, regular blood tests for phenylalanine and other amino acids, regular blood tests for vitamins and micronutrients as prescribed, bone density measurement, brain imaging and neurophysiological testing if needed, genetic testing and counselling and advice for family planning.

Treatment with sapropterin allows a subset of patients to broaden their diet to include more natural protein (some can tolerate a normal diet for the first time after starting sapropterin) and this may improve the quality of life of some patients [11]. Sapropterin treatment should be reimbursed when the importance of improving either metabolic control or decreasing the need of strictness in dietary treatment has been demonstrated. A similar approach applies to new treatments currently in clinical development, once adequate efficacy and safety has been established. Patient organisations, working with healthcare professionals, have an important role here in disseminating reliable information about new treatment modalities to patients and their families.

## Conclusions

There is a clear need to achieve greater uniformity in management practices for patients with PKU across Europe, involving both a greater focus on evidence-based care and also greater involvement of patients, consistent with modern concepts of concordance between patients and physicians [47]. To achieve this, the E.S.PKU is currently working with expert healthcare professionals involved in the management of PKU to produce the first pan-European, evidence-based guideline for the management of PKU. This guideline will establish a minimum standard of care that should become achievable in all countries through uniform access to expert multidisciplinary care, and to dietary and/or pharmacologic treatments. When the guideline is available, we will remain committed to supporting our national member associations to encourage healthcare professionals to adopt and implement the new European guideline to achieve a better and more consistent quality of care for all European PKU patients.

It is crucial that the patient’s perspective is central to the development of these guidelines, as we have set out in this article. Experience in other therapeutic areas has demonstrated the benefits of including a patient perspective in developing management guidelines for chronic, non-communicable diseases [48]. The E.S.PKU urges PKU healthcare professionals caring for people with PKU world wide to take the lead in developing evidence based guidelines on PKU, while continuing to play an active role in serving as the voice of patients and their families, whose lives are affected by the condition.

## Abbreviations

PKU: Phenylketonuria; E.S.PKU: European society for phenylketonuria and allied disorders; PAH: Phenylalanine hydroxylase; HPA: Hyperphenylalaninaemia; BH4: Tetrahydrobiopterin; CE: Centre of excellence; EU: European Union.

## Competing interests

The authors declare that they have no competing interests. Editorial assistance was provided by Dr Mike Gwilt, GT Communications, and Kathleen Duclos, Weber Shandwick, both funded by Merck Serono, after production of initial drafts by the authors. This review was supported by an unrestricted educational grant from Merck Serono.

## Authors' information

The authors represent the E.S.PKU and/or national associations representing people with PKU and their families in European countries. The E.S.PKU is the European umbrella organisation of national and regional PKU patients associations from 25 European countries, acting as a voice for their common interests and the interests of the patients and their families throughout Europe.

## Authors’ contributions

All authors contributed to the writing and critical reviewing of this article. All authors read and approved the final manuscript.
